# Dimethyl Sulfide is a Chemical Attractant for Reef Fish Larvae

**DOI:** 10.1038/s41598-017-02675-3

**Published:** 2017-05-31

**Authors:** Matthew A. Foretich, Claire B. Paris, Martin Grosell, John D. Stieglitz, Daniel D. Benetti

**Affiliations:** 1 Department of Ocean Sciences, Rosenstiel School of Marine and Atmospheric Science, University of Miami, Miami, FL 33149 USA; 2 Department of Marine Biology and Ecology, Rosenstiel School of Marine and Atmospheric Science, University of Miami, Miami, FL 33149 USA; 3 Department of Marine Ecosystems and Society, Rosenstiel School of Marine and Atmospheric Science, University of Miami, Miami, FL 33149 USA

## Abstract

Transport of coral reef fish larvae is driven by advection in ocean currents and larval swimming. However, for swimming to be advantageous, larvae must use external stimuli as guides. One potential stimulus is “odor” emanating from settlement sites (e.g., coral reefs), signaling the upstream location of desirable settlement habitat. However, specific chemicals used by fish larvae have not been identified. Dimethyl sulfide (DMS) is produced in large quantities at coral reefs and may be important in larval orientation. In this study, a choice-chamber (shuttle box) was used to assess preference of 28 pre-settlement stage larvae from reef fish species for seawater with DMS. Swimming behavior was examined by video-tracking of larval swimming patterns in control and DMS seawater. We found common responses to DMS across reef fish taxa - a preference for water with DMS and change in swimming behavior - reflecting a switch to “exploratory behavior”. An open water species displayed no response to DMS. Affinity for and swimming response to DMS would allow a fish larva to locate its source and enhance its ability to find settlement habitat. Moreover, it may help them locate prey accumulating in fronts, eddies, and thin layers, where DMS is also produced.

## Introduction

The early life history of most reef fishes is a multi-stage process during which they are capable of long-distance dispersal; however, the behaviors influencing dispersal are poorly understood. Immediately post-hatch, a larva possesses weak sensory capabilities and limited mobility, thus its transport is presumably passive^1^. As the larva develops it swims in an active^2^ and directed^3^ manner, utilizing its impressive swimming and sensory abilities^4^. Furthermore, biophysical models of larval transport show that the behaviorally-mediated swimming during this stage increases local retention and enhances overall settlement success^5, 6^. As a result, studying the complex behavioral responses of larvae to their sensory environment has emerged as a major component of understanding marine dispersal and population connectivity^4^, focusing on chemical^7–11^, auditory^12, 13^, and celestial^14, 15^ stimuli.

There is strong physiological and evolutionary evidence to suggest that olfaction is the most primitive and universal sense, and that it is intricately linked to odor-tracking and the use of a cognitive odor map^16, 17^. It is not always clear whether olfaction (e.g., chemoreception at the olfactory epithelium) or gustation (e.g., chemoreception on internal or external taste buds) is at work, but both chemosensory systems are well developed in fishes^18^. There is a long history of research on settlement-inducing chemoreception in marine invertebrates^19^, as well as on chemically-induced settlement in fish larvae^20^. Several marine fishes have even evolved the capacity to utilize distinct chemical signals at impressively large spatial scales, specifically salmon^21, 22^ and sharks^23–26^. Nonetheless, little is known about what chemicals play an important role, how perception of these chemicals affects swimming patterns, or mechanisms for their effective use in navigation^27^.

The literature suggesting that larval fish also utilize chemosensory systems for large scale navigation continues to grow. Researchers have hypothesized that “tidal halos” extending several kilometers from settlement sites, which are rich in chemical compounds carried from the reef, may provide important cues for navigation and settlement at varying spatial scales^7^. Population genetic substructure provides additional support for this hypothesis^28^, as does the activation of oriented swimming when larvae enter such a tidal plume^10^. Also, like salmon, some coral reef fish appear capable of imprinting as early as embryonic development and into the first 24 hours post-hatch^29^. The expression of this imprinting seems to vary throughout ontogeny, with chemical preference sharply transitioning from that of offshore waters to that of settlement habitat in one case^30^. It seems very clear that settlement-stage larvae of many reef fishes can and do discriminate chemical environments, preferring those of appropriate settlement habitat and of conspecifics^9, 20, 31, 32^. However, despite this breadth of literature, there is only a weak understanding of exactly what chemicals appeal to larvae (e.g., plant or coral “leachate”).

A logical candidate for a specific chemical involved in larval fish navigation is dimethyl sulfide (DMS), a particularly well-known “infochemical” in the marine environment. DMS has been well studied in the scientific community as far back as 1980’s for its potential role in climate regulation^33^. Since then it has gained attention in the biological community for its role as a chemical attractant^34^. And while most of this work has focused on marine air-breathers such as seabirds^35, 36^, penguins^37^, turtles^38^, and seals^39^, recent work has also demonstrated an affinity in whale sharks^40^. Dimethylsulfoniopropionate (DMSP), a precursor to DMS, has been shown to be a foraging cue for adult reef fishes^41^. Since many larval fishes have highly-developed olfactory systems even before juvenile metamorphosis^42^, it is reasonable to assume that larval fish would have an affinity for similar chemicals.

Moreover, high concentrations of DMS(P) are produced by zooxanthellae^43^, corals^44^, benthic algae^45^, and other coral reef residents. Surface concentrations of DMS(P) are higher near certain coral reefs than anywhere else in the marine environment (as high as nearly 19 μM [DMS] in one area), particularly in the mucus ropes and surface films exported from the reef as a part of the tidal halo^46^. Midwater samples of DMS(P) vary annually, but average from 20 nM to 120 nM^47^, with events such as coral spawning leading to temporal spikes in background concentrations. One further important consideration is that all methods for collecting water samples necessarily lead to an averaging of the DMS(P) concentration over the volume collected. In a chemically heterogeneous environment, chemical concentrations may be much higher at finer spatial scales, and small planktonic organisms are likely to perceive these higher concentrations^48^. In any case, it is clear that, despite considerable temporal and between-reef variation, concentrations are consistently higher around corals than in the pelagic environment. Thus, one would expect that an ability to detect and an affinity for DMS(P) would enhance the recruitment of larval fish at their reef destinations.

In the present study, we utilize a shuttle box (Loligo Systems) adapted to serve as a choice chamber, with DMS-containing seawater on one side and control seawater on the other. We hypothesized that the reef fish larvae would display a preference for the water containing DMS and, further, that their swimming behavior would be altered when they are inside of this water. Experiments were conducted on a range of different reef fish families to determine if observations were consistent for fish larvae with varying ecological traits. Further, in order to assess the relative importance of DMS in indicating reef habitat or food abundance, experiments were also conducted with larvae of mahi-mahi, *Coryphaena hippurus*, a tropical epipelagic fish that spends all its life cycle in the open ocean.

## Results

### Reef Fish Larvae

**Figure 1:**
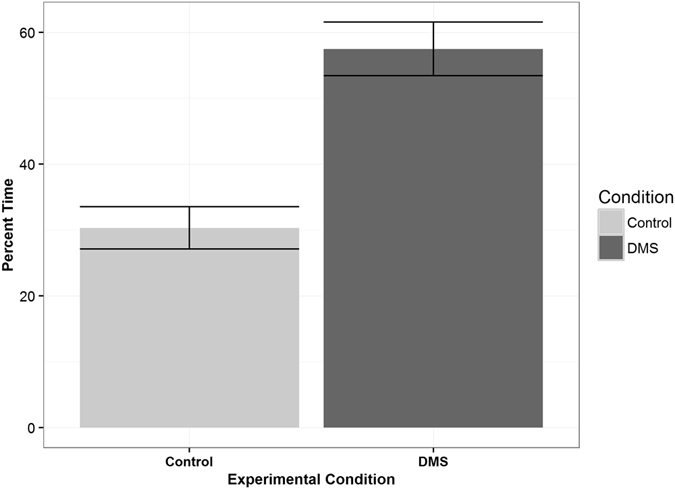
Reef Fish Larvae odor preference. Bars represent one standard error. Pooled together, the 28 larvae tested spent 57.5% of their time in the DMS seawater and 30.3% of their time in the control seawater. The remaining 12.2% of time was spent in the center pathway.

Of the 28 reef fish larvae tested, 23 (82.1%) displayed preference for the side containing DMS, including 16 of the 19 (84.2%) *Stegastes spp* larvae (Table 1). Pooling all trials, including those with a control-side preference, larvae spent 57.5% of their time on the odor side, 1.9x higher than the 30.3% spent on the control side (Fig. 1). The remaining 12.2% was spent inside of the center pathway.

**Table 1:** List of Reef Fish Larvae and their Odor Preference.

Fish Taxa	Sample Size	Odor Preference
*Stegastes partitus*	11	10/11
*Stegastes diencaeus*	7	5/7
*Stegastes variabilis*	1	1/1
*Pomacentridae* (other)	1	1/1
*Hippocampus* spp.	1	1/1
*Serranidae*	1	1/1
*Balistidae*	2	1/2
*Blenniidae*	2	2/2
*Lutjanidae*	1	1/1
*Chaetodontidae*	1	0/1
**Total**	**28**	**23**/**28** (**82**.**1%**)

**Figure 2:**
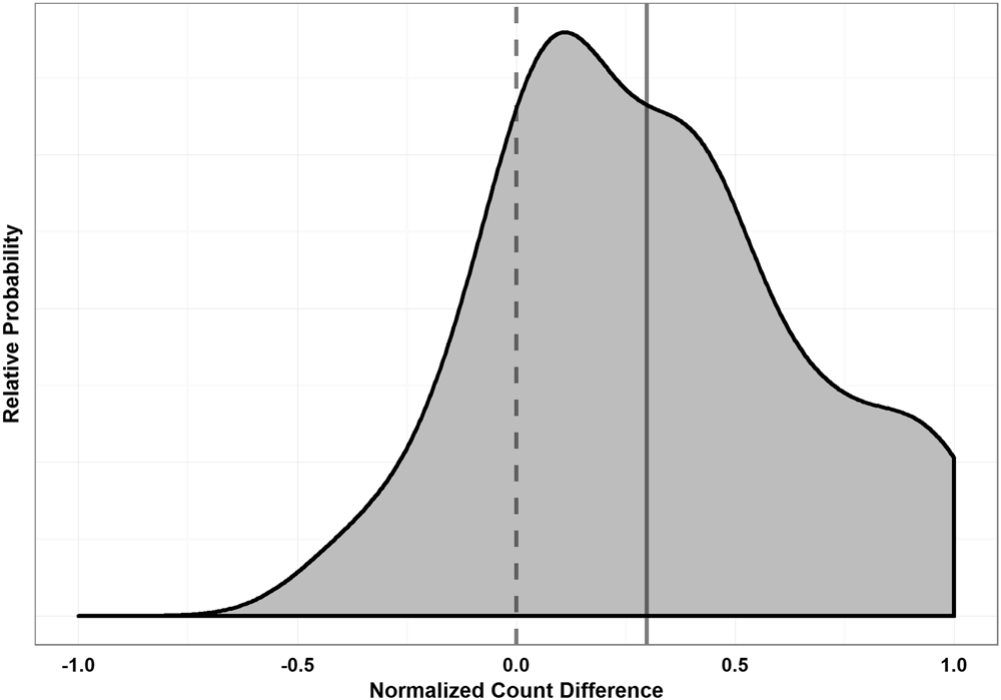
Distribution of Normalized Count Differences (Reef Fish Larvae). Count differences were defined as the number of positional observations on the DMS side minus the number of positional observations on the control side. This was normalized (removed counts from center pathway and divided count difference by total number of remaining observations) to account for the fact that larvae spent varying amounts of time in the center pathway, and thus have different total numbers of positional records in the two chambers. The solid line indicates the data mean, and the dashed line indicates the expected mean under the assumption of no side preference. The mean of the data was significantly greater than 0 (n = 28, α = 0.05, p < 0.0001, t = 4.53, df = 27, one-tailed one-sample t-test), indicating a preference for water containing DMS.

The normalized count-difference variable was used to statistically asses the group-level preference for DMS odor. Figure 2 shows the relative probability distribution of this variable. The right-shifted mean and right-skewed distribution indicates that there was indeed a group preference for DMS odor, and this was statistically significant (n = 28, α = 0.05, p < 0.0001, t = 4.53, df = 27, one-tailed one-sample t-test). Data met assumptions of normality and independence.

**Figure 3:**
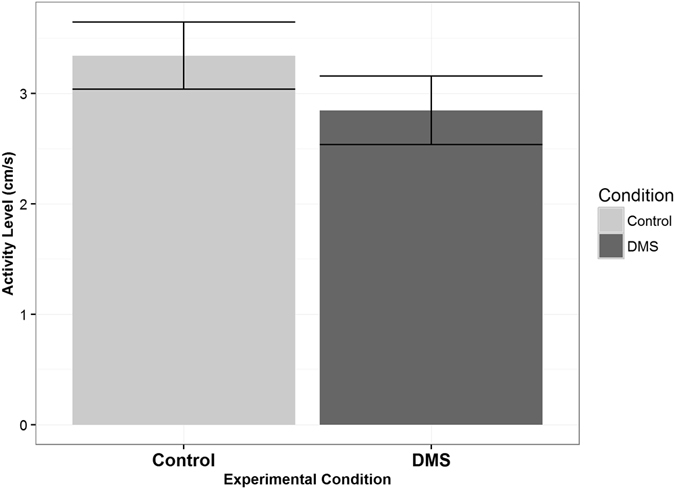
Activity Level of Reef Fish Larvae. Bars represent one standard error. Activity level was defined as the mean of instantaneous velocities calculated from the larval trajectories. The larvae were swimming significantly slower when in the water containing DMS (n = 28, α = 0.05, p = 0.02, t = −2.43, df = 27, paired t-test).

**Figure 4:**
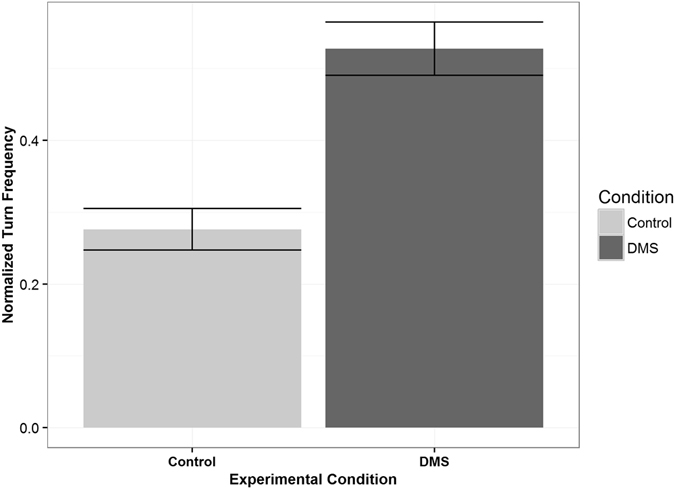
Normalized Turning Frequencies of Reef Fish Larvae. Bars represent one standard error. Turning frequency was defined as the number of turns a larva made which were greater than 45°. This was normalized to account for the fact that, if a larva spent more time on one side than other, this would result in a higher turn frequency on that side without indicating a change in behavior. As a group, larvae made significantly more turns in the odorous water (n = 28, α = 0.05, p = 0.0002, t = 4.29, df = 27, paired t-test).

There were also clear differences in behavior of reef fish larvae when in water containing DMS odor. First, we found differences in the activity level (i.e., mean of instantaneous velocities) of larvae while in odorous water (n = 28, α = 0.05, p = 0.02, t = −2.43, df = 27, paired t-test, Fig. 3); that is, larvae were swimming slower in water containing DMS. Patterns of swimming behavior were also different. Specifically, the normalized turning frequency was significantly higher (n = 28, α = 0.05, p = 0.0002, t = 4.29, df = 27, paired t-test) when larvae were inside of the water containing DMS (Fig. 4). Data met assumptions of normality and independence.

For analysis of group-level behavior, the 28 reef fish larvae of varying taxa were pooled. We justify this decision by the apparent consistency in preference for DMS across taxa. Statistics and complementary power analyses were conducted for the most common species, *S*. *partitus*; the most common genus, *Stegastes spp*; as well as the remaining taxa pooled (see Supplementary Figs S1, S2 and S3). We found statistical significance for all groups at even the α = 0.01 level (see Supplementary material). Further, we did a second analysis extracting only the mean value of the normalized count difference from each of the taxa tested (listed in Table 1). In this way each of the groups was weighted evenly. Results were statistically significant and consistent with the pooled data (Supplementary Figs S4, S5 and S6).

Finally, post-analysis inspection of the video data revealed some common patterns in the behavior of larvae while inside and outside of the odor (Video S7). While in control seawater, larvae tended to display either: (1) swimming directly from the center pathway to the opposite wall of the control chamber, making a 180° turn, and returning to the center pathway or to the other chamber; (2) swimming in a circular motion along the inner circumference of the control chamber. When in the chamber containing DMS odor, larvae displayed a zig-zagging behavior, a characteristic pattern of “search behavior”^49, 50^.

### Mahi-mahi Larvae

Of the 23 mahi-mahi larvae tested, 14 (60.8%) displayed preference for the side containing DMS. Pooling all trials, including those with a control-side preference, larvae spent 56.2% of their time on the odor side, 1.3x higher than the 42.7% spent on the control side. The remaining 1.1% was spent inside of the center pathway.

**Figure 5:**
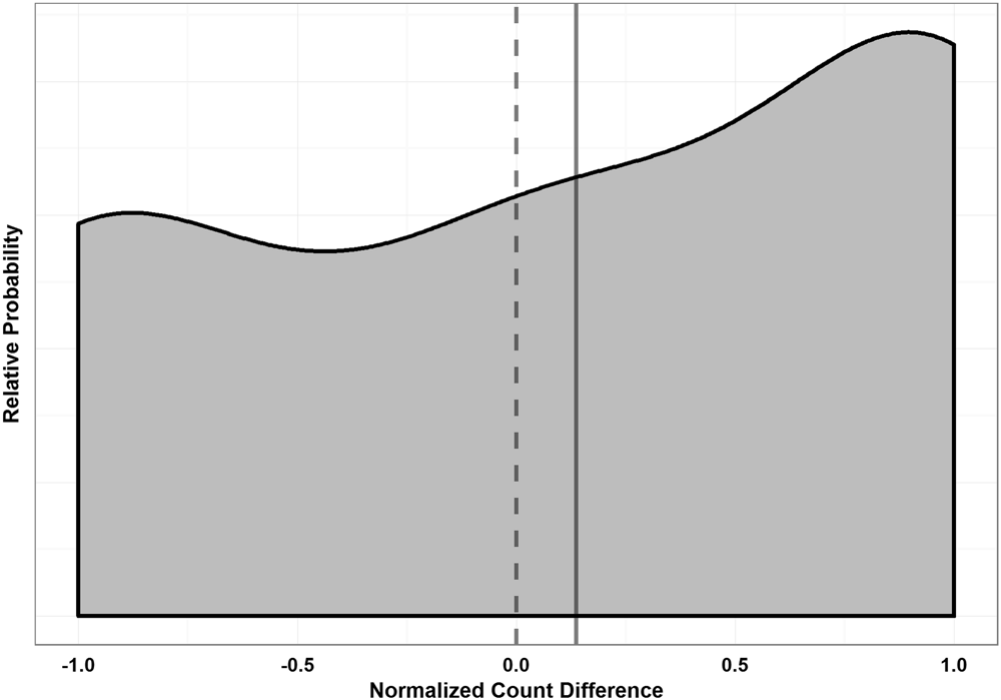
Distribution of Normalized Count Differences (Mahi-mahi Larvae). Count differences were defined as the number of positional observations on the DMS side minus the number of positional observations on the control side. This was normalized (removed counts from center pathway and divided count difference by total number of remaining observations) to account for the fact that larvae spent varying amounts of time in the center pathway, and thus has different total numbers of positional records in the two chambers. The solid line indicates the data mean, and the dashed line indicates the expected mean under the assumption of no side preference. The distribution of the data did not suggest group-level odor preference (n = 23, V = 161.5, α = 0.05, p = 0.24, Wilcoxon signed rank test).

**Figure 6:**
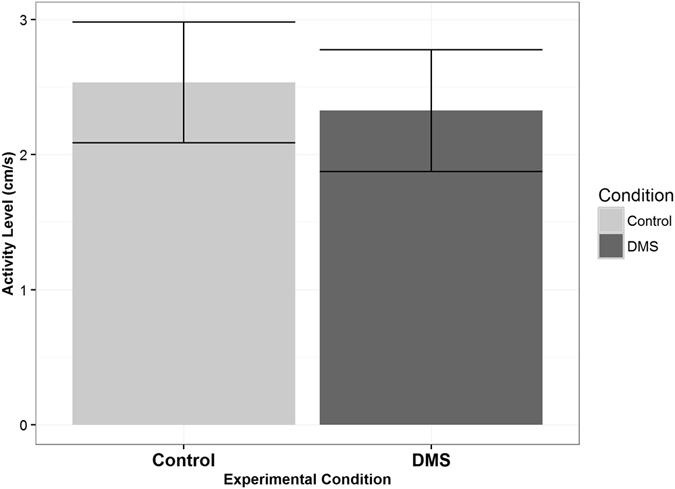
Activity Level of Mahi-mahi Larvae. Bars represent one standard error. Activity level was defined as the mean of instantaneous velocities calculated from the larval trajectories. There was no significant difference in the activity level on the two sides of the shuttle box (n = 23, V = 84, α = 0.05, p = 0.188, Wilcoxon signed rank test).

**Figure 7:**
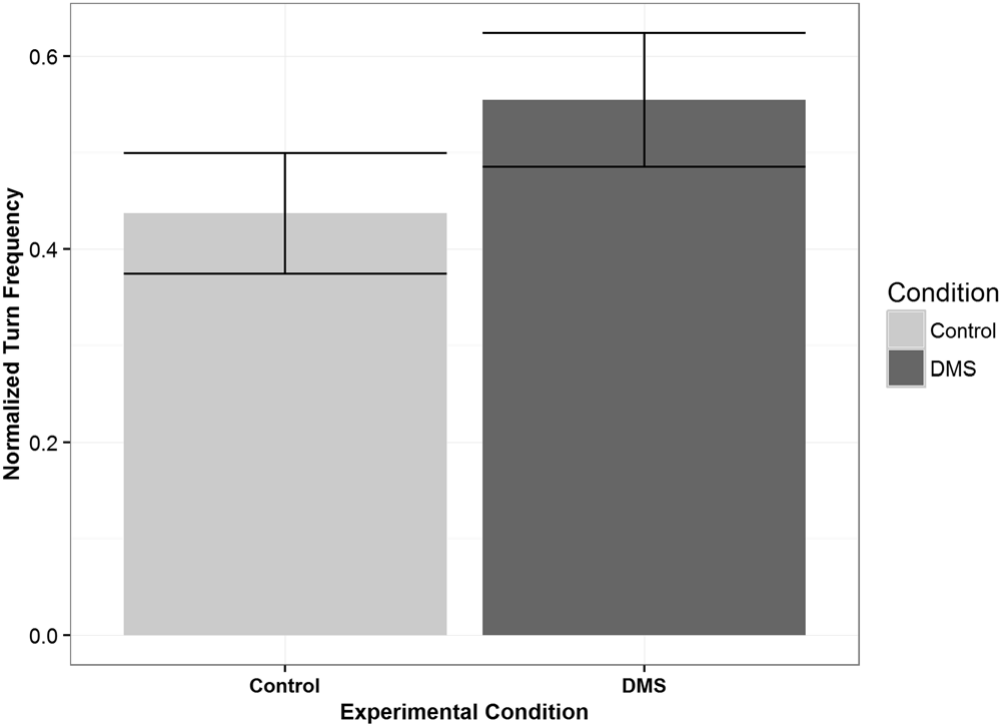
Normalized Turning Frequencies of Mahi-mahi Larvae. Bars represent one standard error. Turning frequency was defined as the number of turns a larva made which were greater than 45°. This was normalized to account for the fact that, if a larva spent more time on one side than other, this would result in a higher turn frequency on that side without indicating a change in behavior. As a group, larvae did not make significantly more turns in the odorous water (n = 23, V = 68, α = 0.05, p = 0.6788, Wilcoxon signed rank test).

The normalized count-difference variable was used to statistically asses the group-level preference for DMS odor. Figure 5 shows the relative probability distribution of this variable, which does not indicate a significant group-level preference for DMS (n = 23, V = 161.5, α = 0.05, p = 0.24, Wilcoxon signed rank test). There were also no differences in swimming behavior between mahi-mahi larvae in control seawater versus DMS containing water. Both activity level (n = 23, V = 84, α = 0.05, p = 0.188, Wilcoxon signed rank test, Fig. 6) and normalized turning frequency (n = 23, V = 68, α = 0.05, p = 0.6788, Wilcoxon signed rank test, Fig. 7) were similar in control water and water containing DMS. Nonparametric statistics were used, as the tested variables were not normally distributed.

## Discussion

We demonstrate that reef fish larvae, across taxa, display a preference for seawater containing DMS. Furthermore, the presence of DMS in the water elicits a different swimming behavior than water without DMS, causing them to swim slower and make larger turns more frequently, consistent with observations in Paris *et al*.^10^ when larvae were inside of tidal halos. We did not, however, observe any preference for DMS in larvae of the epipelagic mahi-mahi, nor did we observe any change in behavior.

The conclusion that reef fish larvae generally possess an affinity for DMS is a significant one. Previous studies have been successful in demonstrating a preference for a chemical suite^7, 10, 28^ or in showing that a single species prefers a particular “leachate”^9^, but this is the first example of cross-taxon reef fish preference for a specific isolated compound. Over the past decade there is an impressive amount of literature displaying the potency of DMS as a universal marine infochemical^35–41^, and this study marks the addition of reef fish larvae to the growing list of attracted organisms.

This study also marks the first use of a shuttle box to assess chemical preference of an aquatic organism. Typically, preference experiments with larval fish have utilized a two-channel flume^7, 11, 28^. The primary benefit of this new method is the ability to simultaneously test the appeal of (or aversion to) a chemical and its effect on the movement patterns of a larva. In this way we are able to assess response to the odor in addition to preference for it. There are, however, a few important remarks regarding the use of a shuttle box for this application: (1) often larvae spend a considerable amount of time in the center pathway connecting the control seawater and the DMS containing seawater. This is likely due to the reduced flow in this region, something which, under stress, may be more important than chemosensory appeal; (2) certain larvae are unsuitable for testing with the shuttle box. Some Ostraciidae and Tetraodontidae larvae, for example, simply did not move. These larvae would stay on the side of the shuttle box they were placed in until the experiment ended, regardless of the length of the experiment. Similar behavior has been observed in subadult *Lutjanus griseus* inside of a shuttle box designed to investigate salinity preference^51^. Some Gerreidae and Clupeidae larvae, on the other hand, would energetically swim into the nearest wall as soon as they were placed into the shuttle box. This places a restriction on which taxa one can reasonably test using this device. We suggest this is also the case for other methods investigating chemical preference in larvae, based on personal communications, and that this is generally underreported. (3) Nearly all larvae spent some time in the control water, despite their significant preference for DMS water. This is likely a result of odor-induced search behavior, whereby the larvae were incited to find the source.

Like other organisms with DMS-affinity, fish larvae may use the presence of DMS as an indicator for nearby food sources^34^. Specific to fish larvae, high concentrations of DMS also suggest the nearby presence of suitable settlement habitat. It is unclear which of these two functions facilitated the evolution of DMS-preference, but it is noteworthy that the concentrations of DMS are higher in coral reef exports (i.e., mucus ropes and surface slicks) than anywhere else in the marine environment, and that the background concentrations are particularly low elsewhere in the tropics^45, 46^. For example, mucus ropes may contain anywhere from 60 to over 12000 times higher DMS concentrations (132 nM–17909 μM) than background (2.2 nM), suggesting DMS may be more useful in finding the reef than in finding prey. Our results here are also telling. While reef fish larvae displayed a strong preference for DMS, mahi mahi did not. This is not surprising, as DMS levels are substantially lower in the epipelagic environment than near reefs.

However, it is also possible that DMS actually serves both roles, either simultaneously or at different points in development of the larvae through its pelagic duration. Indeed, DMS is actually a product of the degradation of DMSP, a chemical which several species of adult fish have a documented preference for^41^. It is unclear whether DMS or DMSP is the “more potent attractant”, if they serve different roles, or if the ratio of the two chemicals is in some way important information for early or late stage larvae.

The idea of a widely-appealing chemical is relatively new and needs to be discussed in the context of perhaps the longest-standing hypothesis in the chemical ecology of larval fish: the imprinting hypothesis. There is strong evidence, both physiological^29^ and behavioral^52^, that supports the idea of chemical imprinting early in fish development, and this study does not intend to refute those findings. Indeed, imprinting on specific microhabitat odors likely plays a very important role in the selection of settlement habitat at a local scale. We speculate that DMS, which is more ubiquitously present at a reef, and would be more broadly exported from it, may be more important as a large-scale infochemical. However, we point out that damselfish of the *Stegastes* genus made up the bulk of our sample, demonstrating that benthic spawners, those most likely to imprint, still display an affinity for DMS. It is possible that DMS, despite its ubiquitous presence on the reef, is actually implicated in the process of imprinting, either in isolation or as one of a suite of chemicals.

This study is an important step forward in understanding the chemosensory navigation of fish larvae and allows us to confidently pursue additional questions. For example: (1) what is the threshold of detection for DMS? or (2) what is the mechanism for navigating using odor plumes? Clear evidence of chemical preferences certainly suggests a mechanism exists, but its nature is unknown. The spatial and temporal distributions of chemical concentrations are notoriously complex and composed of highly-concentrated filaments interspersed with fluid containing little or no chemical^53^. The structure depends on the dominant flow, turbulent diffusion, topography, etc.^48^ and creates a serious problem for traditional gradient-based odor-tracking strategies. Knowing the threshold of detection for DMS will allow observations of behavior to be compared to models of behavioral responses.

Which strategies are being used by minute fish larvae to find their settlement sites from the open ocean remains a mystery. Future studies should use DMS, at relevant concentrations above threshold of detection, to simultaneously record odor plume structure and larval fish kinematics. This will provide valuable insight on the mechanism being used to find the source of the odor. The identification of DMS as a universal infochemical for larval reef fish brings us closer to realizing this goal.

## Methods

### Study Organisms

Larval fish used in the experiment were obtained by one of two ways: (1) Wild-caught utilizing light traps, or (2) Reared in the University of Miami Experimental Hatchery (UMEH) at the Rosenstiel School of Marine and Atmospheric Science. Light traps (CARE, EcoOcean; BellaMare, LLC) were set on fore reef off the coast of Key Biscayne, FL, USA (25°70478N; 80°09790W and 25°67410N; 80°09420W). Traps were set at dusk and fish were retrieved from traps at dawn. All experiments were conducted within 24 hours of larval collection, and all larvae were drip-acclimated to the water from the seawater system in the Marine Technology & Life Sciences Seawater Research Building at University of Miami’s Rosenstiel School. Identifications were made using morphological key characteristics^54^ and incidental mortalities allowed certain specimens to be saved for detailed taxonomy. All other individuals were freed in the nearby bay following their behavioral trial, in accordance with the guidelines of University of Miami IACUC protocol #14-216. All methods were carried out in accordance with relevant guidelines and regulations, defined in said protocol, approved by University of Miami’s Institutional Animal Care and Use Committee. A total of 28 larvae belonging to 10 taxa were caught and used in experiments, (Table 1). The two most common larvae were damselfish belonging to the *Stegastes* genus, *Stegastes partitus* (n = 11) and *Stegastes diencaeus* (n = 7). All larvae were wild-caught opportunistically from light-traps, and thus experiments were conducted on a wide array of fish families. Only one genus of damselfish, *Stegastes*, had substantial representation in our dataset (n = 17).

Mahi-mahi larvae were reared from fertilized eggs obtained from volitional spawning wild mahi-mahi broodstock maintained at the UMEH facility. Larval rearing protocols were similar to those described by Benetti *et al*.^55^ and Kraul (1993)^56^ for rearing of cobia (*Rachycentron canadum*) and mahi-mahi, respectively. Eggs were hatched and larvae were reared in 2.4 m^3^ cylindrical fiberglass tanks. Beginning on day 3 post hatch the rearing water was inoculated with *Nannochloropsis* microalgae paste (Reed Mariculture Inc.) at a level to maintain a slight green color in the water column (~250,000 cells mL^−1^). Enriched rotifers (*Briachionus plicatilis*) and *Artemia* nauplii were fed to the larvae over the course of the rearing period, and water exchange averaged 10 turnovers per day in the rearing tanks. Larvae used for this study were randomly removed from the larval rearing tanks using a submerged 1-L beaker to avoid physical contact and they were subsequently held in 15-L vessels containing UV-sterilized aerated seawater in the hours leading up to testing.

### Shuttle box System

**Figure 8:**
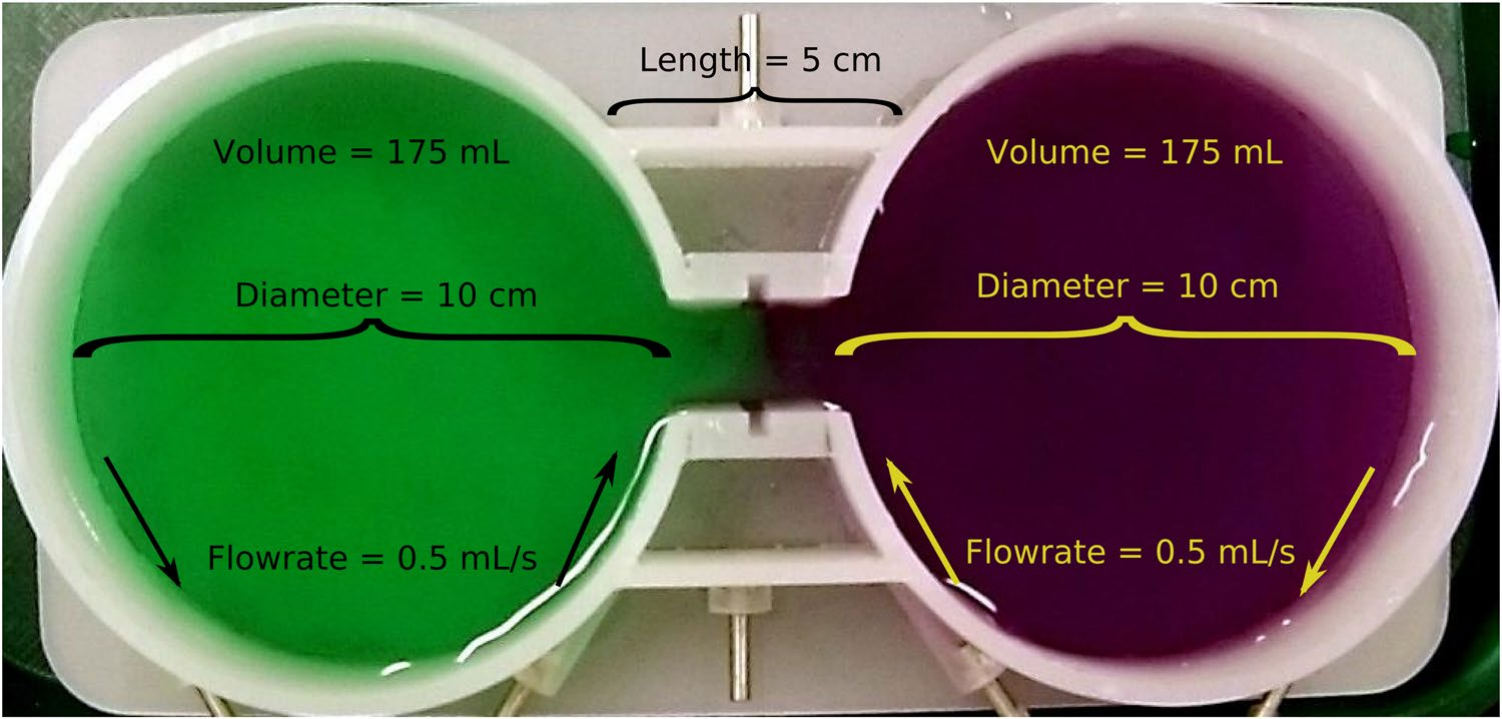
Shuttle-box Dimensions and Dye Test. Each of the chambers has a 10 cm diameter and holds approximately 175 mL of water when filled to the desired height. The flow rates are set to 0.5 mL/s for both inflows and outflows, and the inflows are always at the most centrally-positioned ports. The 5 cm distance at the top is used during the tracking process to calibrate pixels to cm for realistic instantaneous speed calculations. The coloration of the sides is the result of a 20 minute dye-test where the inflows for each side are either green or purple. There is very little mixing between the two sides, and the flow-through design of the system reduces accumulation of mixing.

A novel methodology utilizing a shuttle box design (Loligo Systems, Fig. 8) was used in order to assess larval chemical preference. The system consists of two 10 cm diameter circular chambers connected by a short (<3 cm) narrow pathway allowing free movement of a larva. Each of the chambers is fitted with two tangential ports to attach inflow and outflow tubing. This positioning and the low flow rate (0.5 mL/s) produce smooth circular flow inside of the chambers and serves to greatly reduce mixing between the two sides. An iPumps i150 peristaltic pump with a B4 R6 four channel, 6 roller pump head (iPumps Limited) was used to ensure precise and equal flow rates at each of the inflows and outflows. Before any set of experiments a dye test was always conducted to ensure that there was no substantial mixing between the two chambers (Fig. 8).

### Experimental Protocol

Odor preference experiments were conducted on one fish larvae at a time and lasted a total of 20 minutes. First, a solution of 340 nM DMS was prepared through serial dilution of a >99% standard (Sigma-Aldrich). Next, the shuttle box was filled with approximately 175 mL of water in each chamber and a larva was randomly introduced to one side. The experiment began when the pump was activated, introducing seawater with 340 nM DMS on side and seawater with 0 nM DMS on the other; sides were chosen at random. 340 nM was chosen as the stock concentration based on the maximum measurement of DMS in sediment pore waters at Kelso Reef, a mid-shelf oligotrophic patch reef in the central Great Barrier Reef^46^. Stock solutions for substantially larger aquatic organisms have varied from 100 nM for whale sharks^40^ and adult reef fishes^41^ *in situ* to 200 nM for adult reef fishes in lab flumes^47^. Our concentration was slightly higher, as a smaller organism should be able to sample higher concentrations at finer spatial scales. Exact ecologically-relevant concentrations are unknown, as there are no studies specifically addressing the spatial/temporal variation of DMS concentrations at varying distances from coral reefs, and we present a best guess at a relevant concentration given our current knowledge. The concentrations of both stock solutions were verified using vapor-generation and chemoluminescence-detection^57^ and a new stock DMS solution was created every other experiment to account for the naturally declining concentrations of DMS in solution over time.

The 20 minutes of each trial was split into 5 minutes of acclimation and 15 minutes of experiment. The acclimation period allowed the larvae to adjust to their new environment and also for the concentration of DMS to accumulate inside one of the chambers (Supplementary Fig. S6); at 5 minutes the concentration inside the chamber is roughly 200 nM, representing the mean concentration of the benthos near Kelso Reef^46^. No one was permitted to enter the experimental room during these 20 minutes, and the movement of the larva inside of the shuttle box was recorded by a video camera set to 30 frames per second. At the end of the trial the larva was removed, the shuttle box and tubing was thoroughly rinsed with ethanol and seawater, and the sides for larval introduction and odor were randomly chosen for the next experiment.

### Movement Analysis

Video data was subsampled using VirtualDub (version 1.10.4, http://www.virtualdub.org) to record the position of the larva inside the shuttle box at 1 second intervals. Custom software was designed integrating R (R Core Team 2014, version 3.1.2, R Foundation for Statistical Computing, http://www.R-project.org) and ImageJ (W.S. Rasband, version 1.46r, NIH, Bethesda, MD; http://rsb.info.nih.gov/ih/) to process each image sequence. Briefly, the user manually selected the boundary of both chambers, used a portion of the shuttle box with known length to calibrate pixels to centimeters, and then selected the approximate position of the larva’s head in every frame. The program used these positional records to determine the amount of time spent in each chamber, instantaneous velocities, and turning angles. Records of larval position in the center pathway were excluded from analysis.

### Statistical Analysis

A second level of statistical analysis was performed in R to examine group behavioral trends on both sides of the shuttle box. First, we investigated the average amount of time spent on the two sides of the chamber. Next, we calculated the “count difference”, which is, for each individual trial, the number of positional records in the odorous chamber minus the number of positional records in the control chamber. This count difference variable, if there is no overall preference for the odor, should be normally distributed with a mean of 0. A one-sample t-test (α = 0.01) was used to test the hypothesis that the mean was indeed equal to 0 when data met the assumptions of normality, independence, and random sampling. For data that was not normally distributed, a nonparametric Wilcoxon signed rank test was used. Because positional records where the larvae is in the center pathway were excluded, this variable was normalized to account for the different number of records between trials (i.e., some larva spend more time in the center pathway than others). This was accomplished by removing those records from the dataset and dividing the count difference by the number of records remaining.

In addition to side preference, behavioral differences while in each side of the chamber were assessed. Specifically, the mean swimming speed (which, for each trial, is the mean of the instantaneous speeds) and the variation in swimming speed were compared. The frequency of large turns (i.e., greater than 45°) was also compared. This latter variable was normalized to account for the fact that larva may have spent more time on one side than another, increasing the frequency of turns on that side without indicating a change in behavior. This was accomplished by dividing the turning frequency on each side by the total number of records on that side. A paired t-test (α = 0.01) was used to test the hypothesis that mean turning frequencies and activity levels were equal in seawater containing DMS and control seawater when our data met assumptions of normality, independence, and equal variance between groups, otherwise a nonparametric paired Wilcoxon signed rank test was used. Finally, while tracking was done without knowledge of the odorous side, a qualitative assessment of swimming behavior on each side was used to explain quantitative findings.

## Electronic supplementary material

Supplementary Information

Supplementary Video
