# Supplementary material for: Dimethyl Sulfide is a Chemical Attractant for Reef Fish Larvae
Source: Sci Rep. 2017 May 31;7:2498. doi: 10.1038/s41598-017-02675-3 (PMC5451384; doi:10.1038/s41598-017-02675-3)
Supplement: Supplementary file 1 — Supplementary Information [file 41598_2017_2675_MOESM1_ESM.pdf]

**Dimethyl Sulfide is a Chemical Attractant for Reef Fish Larvae**

Matthew A Foretich[1*], Claire B Paris[1], Martin Grosell[2], John D. Stieglitz[2], and Daniel D Benetti[2]

[1]Department of Ocean Sciences, Rosenstiel School of Marine and Atmospheric Science, University of Miami, Miami, FL, 33149, USA

[2]Department of Marine Biology and Ecology, Rosenstiel School of Marine and Atmospheric Science, University of Miami, Miami, FL, 33149, USA

* Corresponding Author
E-mail: mforetich@rsmas.miami.edu

# SUPPLEMENTARY MATERIAL

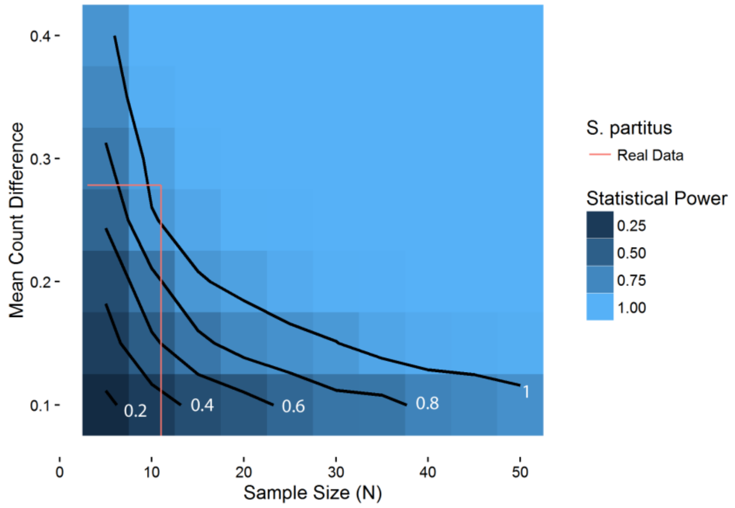


Supplementary Figure S1. Power Analysis for *S. partitus* odor preference. Because of low sample size for this species, we investigated statistical power. We produced a maximum likelihood estimate of the mean (0.278) and standard deviation (0.281) of the count difference variable for *S. partitus* (N=11). We then varied the value of the mean around this estimate, keeping the standard deviation constant, while also varying the number of samples, to produce hypothetical normal distributions. This was repeated 500 times, each time extracting a p-value from a one-sided one-sample t-test. Our power was, by definition, the number of times the p-value was smaller than 0.05, divided by the total number of simulations (500). The figure shows real data values in red, contours of 0.2 power as black lines, and each tile is represented by a power estimate given in the legend. We conclude that our detected mean and standard deviation are indicative of real preference, and increasing the sample size was unnecessary with the strength of the effect size observed in the data. Note that these data were significant at even an $\alpha$ level of 0.01 ($p = 0.0053$).

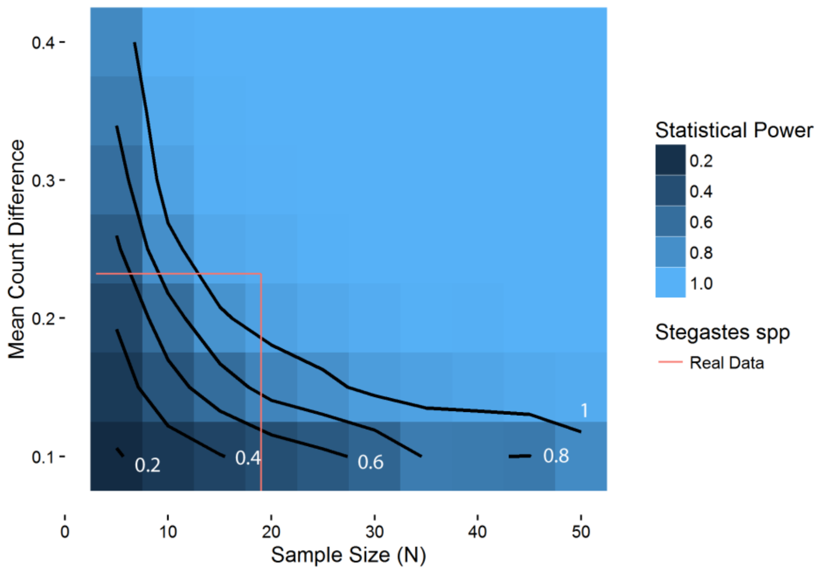


Supplementary Figure 2. Power Analysis for *Stegastes spp.* odor preference. Because of intermediate sample size for this genus, we investigated statistical power. We produced a maximum likelihood estimate of the mean (0.232) and standard deviation (0.294) of the count difference variable for all *Stegastes spp* (N=19). We then varied the value of the mean around this estimate, keeping the standard deviation constant, while also varying the number of samples, to produce hypothetical normal distributions. This was repeated 500 times, each time extracting a p-value from a one-sided one-sample t-test. Our power was, by definition, the number of times the p-value was smaller than 0.05, divided by the total number of simulations (500). The figure shows real data values in red, contours of 0.2 power as black lines, and each tile is represented by a power estimate given in+ the legend. We conclude that our detected mean and standard deviation are indicative of real preference, and increasing the sample size was unnecessary with the strength of the effect size observed in the data. Note that these data were significant at even an α level of 0.01 (p = 0.0018).

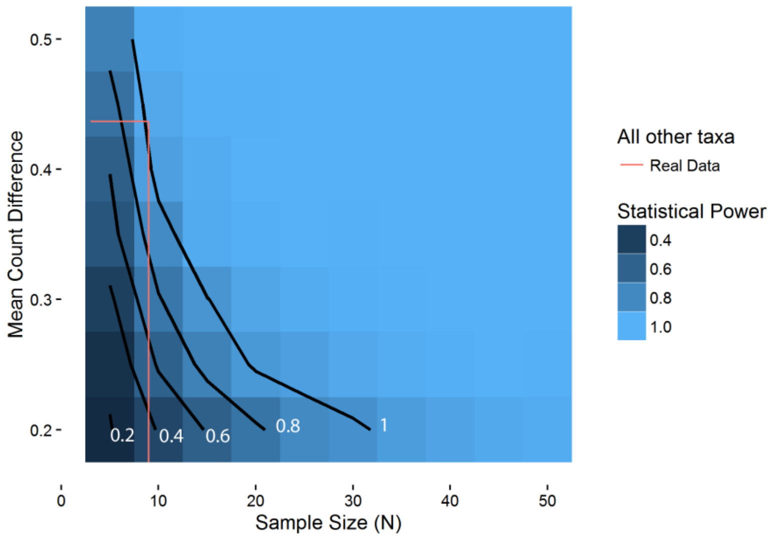


Supplementary Figure S3. Power Analysis for odor preference of all remaining taxa. Because of low sample size for all non-*Stegastes* reef fish larvae, we investigated statistical power. We produced a maximum likelihood estimate of the mean (0.435) and standard deviation (0.39) of the count difference variable for all remaining taxa (i.e., not *Stegastes spp,* N=9). We then varied the value of the mean around this estimate, keeping the standard deviation constant, while also varying the number of samples, to produce hypothetical normal distributions. This was repeated 500 times, each time extracting a p-value from a one-sided one-sample t-test. Our power was, by definition, the number of times the p-value was smaller than 0.05, divided by the total number of simulations (500). The figure shows real data values in red, contours of 0.2 power as black lines, and each tile is represented by a power estimate given in the legend. We conclude that our detected mean and standard deviation are indicative of real preference, as adding additional samples would not have increased statistical power. Note that these data were significant at even an $\alpha$ level of 0.01 ($p = 0.0067$).

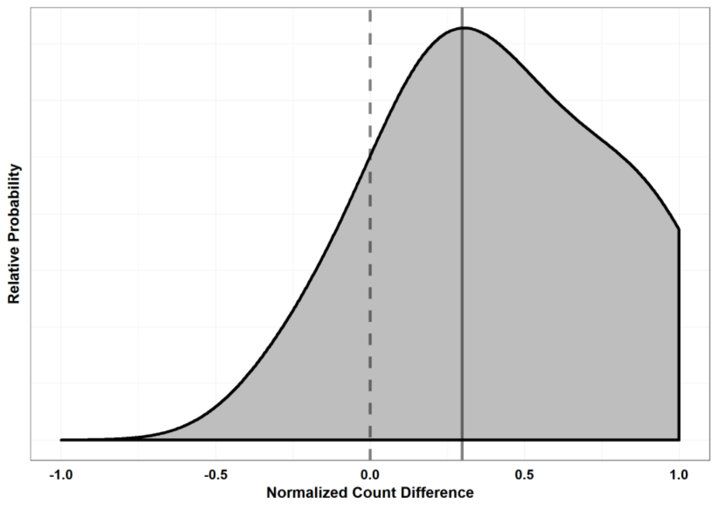


Supplementary Figure S4. Distribution of Normalized Count Differences (Equally-weighted taxa). Count differences were defined as the number of positional observations on the DMS side minus the number of positional observations on the control side. This was normalized (removed counts from center pathway and divided count difference by total number of remaining observations) to account for the fact that larvae spent varying amounts of time in the center pathway, and thus has different total numbers of positional records in the two chambers. The solid line indicates the data mean, and the dashed line indicates the expected mean under the assumption of no side preference. The mean of the data was significantly greater than 0 ($n=10$, $\alpha= 0.05$, $p=0.002$, $t=3.73$, $df=9$, one-tailed one-sample t-test), indicating a preference for water containing DMS.

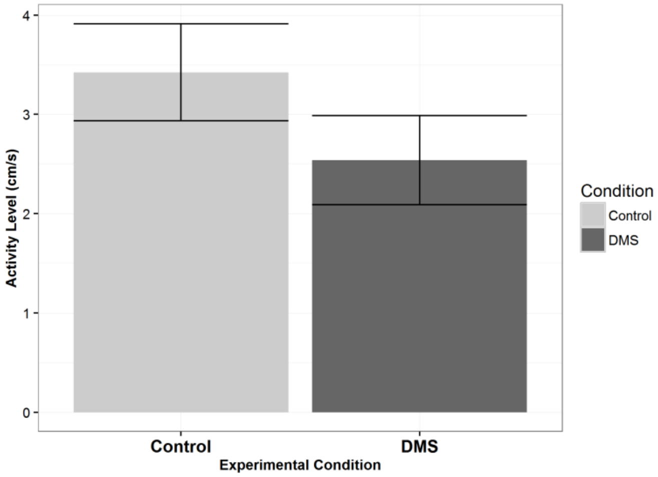


Supplementary Figure S5. Activity Level of Reef Fish Larvae (Equally-weighted). Bars represent one standard error. Activity level was defined as the mean of instantaneous velocities calculated from the larval trajectories. There larvae were swimming significantly slower when in the water containing DMS ($n=10$, $\alpha=0.05$, $p=0.04$, $t= -2.26$, $df=9$, paired t-test).

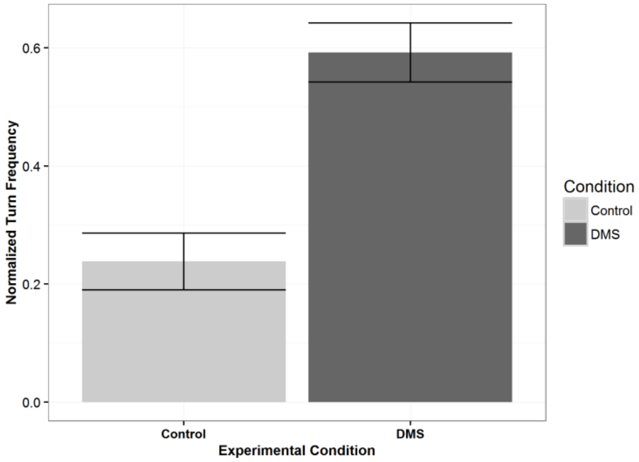


Supplementary Figure S6. Normalized Turning Frequencies of Reef Fish Larvae (Equally-weighted). Bars represent one standard error. Turn frequency was defined as the number of turns a larva made which were greater than 45°. This was normalized to account for the fact that, if a larva spent more time on one side than other, this would result in a higher turn frequency on that side without indicating a change in behavior. As a group, larvae made significantly more turns in the odorous water (n=10, α=0.05, p=0.004, t=3.68, df=9, paired t-test).

[video file submitted separately]

Supplementary Video S7. Characteristic Larval Swimming Pattern. In this trial, the left side of the shuttle box contains DMS. In addition to spending more time on the left side, the larva alters its swimming behavior, taking more turns and zig-zagging through the center of the chamber.

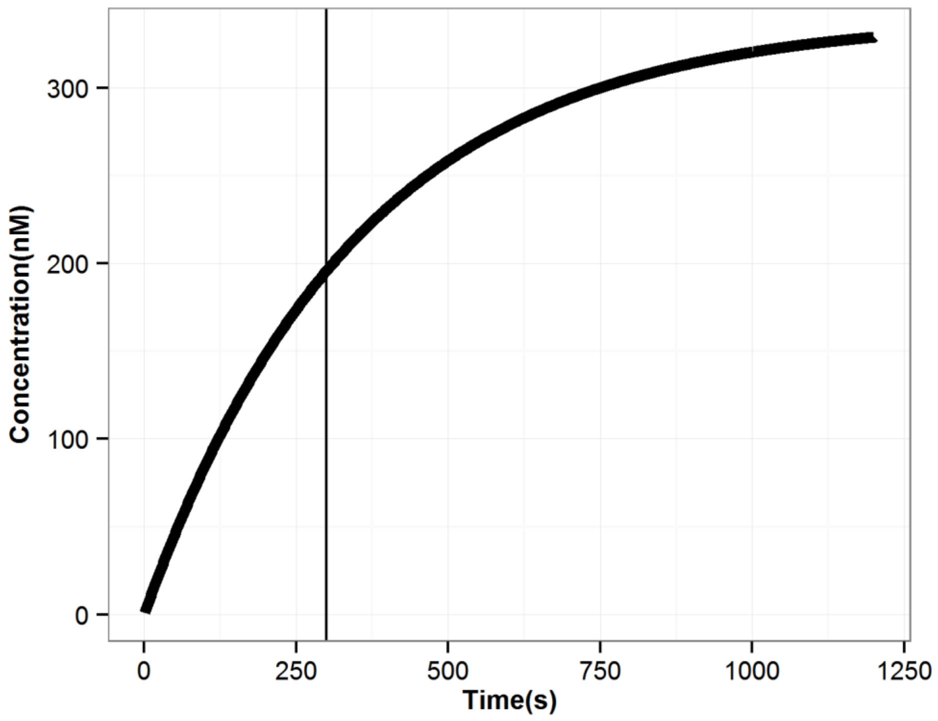


Supplementary Figure S8. Theoretical DMS concentrations during an experiment. The concentration of DMS increases in the odorous chamber over time as stock solution (340 nM) enters through the inflow and water inside the chamber exits through the outflow. The vertical black line indicates the end of the acclimation period (5 minutes), where the concentration of DMS inside the chamber is approximately 200 nM. The concentration at the end of the 15-minute experimental period has nearly reached 340 nM.
